# The Effects of Omega-3 Fatty Acid Supplementation on the Lipid Profile and Cardiovascular Markers Following Downhill Running in Long-Distance Runners

**DOI:** 10.5114/jhk/174107

**Published:** 2023-10-27

**Authors:** Marzena Jaworska, Szymon Siatkowski, Aleksandra Żebrowska

**Affiliations:** 1 Department of Physiological and Medical Sciences, Institute of Sport Science, The Jerzy Kukuczka Academy of Physical Education in Katowice, Katowice, Poland.; 2 Institute of Healthy Living, The Jerzy Kukuczka Academy of Physical Education in Katowice, Katowice, Poland.

**Keywords:** endurance training, erythrocyte content of fatty acids, cardiac damage markers, inflammation, diet

## Abstract

Exercise-induced injury may intensify inflammatory response and reduce the cardiovascular protection mechanisms of omega 3 polyunsaturated fatty acids (ω 3 PUFA). Therefore, this study aimed to determine the erythrocyte content of fatty acids (ω 3 and ω 6), the levels of cardiac damage markers (CKMB, hsTnT, H - FABP), the concentration of inflammation mediators (IL-6, TNF α) in long distance runners supplemented with ω 3 PUFA. Twenty-four male long distance runners, who were randomly assigned to a placebo group (GrP) or a group supplemented (GrSuppl) with a daily dose of 3,000 mg of ω 3 PUFA for three weeks, participated in the study. Participants performed a downhill running exercise test. Blood samples were collected at rest and after the exercise protocol to analyse the levels of cardiac markers and inflammatory cytokines. The erythrocyte membrane content of EPA and DHA in the GrSuppl at the 3^rd^ week of supplementation was significantly higher than at the baseline (p < 0.001). The erythrocyte membrane content of ω 3 PUFA in the GrSuppl was significantly higher at the completion of supplementation (p < 0.001). Supplementation with ω 3 PUFA improved blood lipid profiles and reduced the concentration of inflammation mediators measured after the eccentric exercise tests. The increased ω 3 PUFA content in the erythrocyte membrane and lower blood concentrations of cardiac damage markers and inflammation mediators in distance runners supplemented for three weeks with ω 3 PUFA suggest that the cardiovascular function has been improved.

## Introduction

The observed trend of increasing participation rates in marathon and ultramarathon events indicates popularity of this form of recreational and competitive sport (ARRS—Homepage, https://arrs.run/, accessed on 23 July 2021; Reusser et al., 2021). Numerous studies have shown a variety of health benefits, including the improvement in sport performance and cardiovascular health (Schoenfeld et al., 2020), induction of functional changes in the neuromuscular system and modulation of inflammation (Burkule et al., 2016, Knechtle and Nikoladis 2018). Nevertheless, marathon runners also face health risks (Jastrzębski et al., 2009; Knechtle et al., 2018; Neilen et al., 2006). The risk of lower extremity (hamstring) muscle injury (Higashihara et al., 2020), cardiac dysfunction and cardiac arrhythmia increases with the intensity of running and makes marathon runners prone to sport specific injuries (Neilan et al., 2006; Predel, 2014). Exercise-induced muscle damage results in decrements in muscle force production and an increase in blood cytokines, which contribute to systemic inflammation. This inflammatory response to exercise can be intensified by eccentric components of muscle contraction (downhill running) (Eston et al., 1995).

Recent studies highlight that nutrition is a critical component of the preparatory and competitive phases in long distance running and plays a fundamental role in performance (Costa et al., 2019; Tiller et al., 2019; Martínez-Navarro et al., 2021). One class of nutrients that appears to have anti-inflammatory properties are n-3 polyunsaturated fatty acids (omega 3, ω 3 PUFA), including eicosapentaenoic acid (EPA) and docosahexaenoic acid (DHA). Supplementation with ω 3 PUFA may represent a useful therapeutic agent for attenuating muscle damage (Micklelborough et al., 2015), abating the risk of cardiovascular dysfunction (Abdelhamid et al., 2020; Innes and Calder, 2020), and improving the immunological defence of the body (Buonocore et al., 2020; Oppedisano et al., 2020). It is particularly important that people who engage in straining activities, such as marathon runners, match the supply of dietary ω 3 PUFA. Studies have reported that endurance training reduces the erythrocyte content of ω 3 PUFA, i.e., EPA and DHA (Mickleborough, 2013; Schacky et al., 2014; Thielecke and Blannin, 2020). Additionally, the n-6 polyunsaturated fatty acids (ω 6 PUFA)-derived lipid mediators are released following exercise-induced muscle damage, promoting acute inflammation by modulating the blood flow, vascular permeability, and cytokine synthesis (Davinelli et al., 2019).

Exercise-induced injury may intensify an inflammatory response and reduce the cardiovascular protection mechanisms of ω 3 PUFA (Burillo et al., 2012; Mason et al., 2020; Schacky et al., 2020). Importantly, the protective effect of ω 3 PUFA may be negatively modulated by an increased consumption of saturated fats or ω 6 PUFA (Gammone et al., 2019). A high ratio of ω 6 PUFA arachidonic acid (AA) to EPA (AA/EPA) has been suggested to promote inflammation, and has been proposed as a predictor of sudden cardiac death (SCD) (Hayakawa et al., 2012). The anti-inflammatory properties of ω 3 PUFA may be associated with its ability to reduce the secretion of pro-inflammatory cytokines (i.e., tumour necrosis factor alpha, interleukin 6) and reactive oxygen species through the modulation of neutrophil function (Gutiérrez et al., 2019; Żebrowska et al., 2021).

Previous studies analysing the health risks associated with marathon and ultramarathon running have reported the relationship between the optimal dietary ω 3 PUFA consumption, and its potential implications for sport performance (Buonocore et al., 2021; Costa et al., 2019; Davinelli et al., 2019). Given the above, it seems to be important to determine whether higher dietary ω 3 PUFA consumption protects the cardiovascular system of long distance runners.

Echocardiography and specific cardiac damage markers in response to marathons and ultramarathons have been extensively studied (Khode et al., 2015; Kneble et al., 2009; Rubio-Arias et al., 2020). It has been shown that high-sensitivity cardiac troponin T (hsTnT), creatine kinase isoenzyme MB (CKMB), and fatty acid binding protein (H-FABP) expression increase after a marathon and an ultramarathon race (Kim et al., 2014; Rubio-Arias et al., 2014; Żebrowska et al., 2019). According to the previous data, the biomarker levels typically return to baseline values within 5 to 48 h after exercise (Mahanty and Xi, 2020; Żebrowska et al., 2019). Nevertheless, repetitive increases in the levels of cardiac damage markers and hypoxia may be detrimental to the cardiovascular system in the long term (Kaleta-Duss et al., 2020).

Hypertrophic cardiomyopathy and coronary vessel anomalies are the main risk factors of cardiovascular disease among athletes. Young athletes (<35 years of age) are at a lower risk of cardiovascular disease compared to athletes aged 35 and older, among whom the most frequent cause of SCD is coronary atheromatosis (Predel, 2014; Schoenfeld et al., 2020). Given the above, developing optimal dietary support to protect the cardiovascular system of endurance athletes, marathon and ultramarathon runners in particular, can help reduce their risk of SCD. Although research studies confirm multiple benefits of increased dietary content of ω 3 PUFA (Adbelhamid et al., 2020; Bernasconi et al., 2021; Drobnic et al., 2017), there is little evidence on whether ω 3 PUFA can improve cardiovascular adaptation to strenuous exercise with downhill running components in athletes. Whether these adaptive changes are manifested through an elevated ω 3 PUFA index, thus, increased erythrocyte content of ω 3, has not been determined yet.

Therefore, this study aimed to evaluate the effects of three-week supplementation with ω 3 PUFA extract on the erythrocyte ω 3 PUFA content (HS-Omega 3 Index^®^), the levels of cardiac damage markers (CKMB, hsTnT, H - FABP), and the concentration of inflammation mediators (IL-6, TNF α) in long distance runners after an eccentric exercise protocol (a downhill run). The following hypothesis was formulated: a 3-week supplementation program with ω 3 PUFA would increase the erythrocyte ω 3 PUFA content as measured by HS-Omega 3 Index^®^ and decrease the cardiac damage markers, and inflammation mediators in long distance runners after an eccentric exercise protocol.

## Methods

### Participants

The study involved twenty-four male long distance runners who were randomly assigned either to a placebo group (GrP) (age: 35.9 ± 5.3 years) or a group to receive ω 3 PUFA (GrSuppl) (age: 33.7 ± 7.7). Basic anthropometric measurements and physical performance assessment were conducted in all participants. Their body mass and body composition were determined using the InBody Data Management System (Biospace Inc., Seoul, Korea) (Table 1).

**Table 1:** Characteristics of participants in the supplemented (GrSuppl) and the placebo group (GrP) (mean ± SD).

*Variables*	*GrSuppl* *n = 12*	*GrP* *n = 12*
*Age (years)*	*33.7 ± 7.5*	*35.9 ± 5.3*
*Body mass (kg)*	*74.7 ± 10.3*	*75.3 ± 8.6*
*Body height (cm)*	*176.8 ± 6.0*	*178.2 ± 6.8*
*BMI (kg/m* *^2^* *)*	*23.8 ± 2.2*	*23.7 ± 2.1*
*Fat (%)*	*13.7 ± 3.3*	*13.5 ± 4.4*
*SMM (kg)*	*36.5 ± 5.1*	*36.9 ± 4.5*
*TBW (l)*	*47.2 ± 6.4*	*47.5 ± 5.4*
*VO_2max_* *(ml/kg/min)*	*54.5 ± 9.4*	*58.1 ± 7.4*
Peak power output (watt)	*321.5 ± 77.9*	*351.4 ± 68.3*
HR_max_ (beats per min)	*181.0 ± 11.0*	*186.0 ± 9.0*
Training experience (years)	*10.8 ± 4.1*	*10.3 ± 3.9*

BMI: body mass index, Fat: percent body fat, SMM: skeletal muscle mass, TBW: total body water, VO_2max_: maximal oxygen uptake, HR_max_: maximal heart rate

Athletes ran on average a total of 60–110 km a week and during at least three training sessions per week they ran for at least 15 km. Participants were recruited based on the following criteria:

- voluntary consent to participate in the study,
- at least three years’ experience of distance running,
- completion of at least two marathon races,
- adult age,
- good health and ability to perform eccentric exercise.

The exclusion criteria were as follows:

- medical contraindications certified by a physician,
- an injury or inflammation precluding participation in eccentric exercise tests,
- cardiovascular insufficiency,
- the use of supplements and/or anti-inflammatory medications within at least two months prior the study; this exclusion criterion also applied to participants who would use such substances outside the nutritional protocol during the study period,
- the use of WADA-banned substances,
- smoking cigarettes,
- a running experience shorter than three years.

Three weeks before supplementation, runners consumed an individually prepared diet with the same amount of energy (35 kcal per kg of body mass per day). Daily carbohydrate (48.1 ± 6.3% vs. 49.0 ± 7.5%), fat (28.6 ± 7.6% vs. 28.8 ± 7.3%) and protein (22.8 ± 5.4% vs. 22.4 ± 3.3%) intake was comparable in the GrSuppl and the GrP (Tiller et al., 2019). Daily saturated fatty acid (SFA) and monounsaturated fatty acid (MUFA) intake was also comparable in the GrSuppl and the GrP (SFA: 37.4 ± 15.2 vs. 33.5 ± 9.6 g/d, respectively, and MUFA: 29.6 ± 10.1 vs. 26.0 ± 8.7 g/d, respectively). The same dietary regimen was maintained over three weeks of supplementation, during which participants took ω 3 PUFA or a placebo depending on their group assignment.

All participants were asked to complete weekly questionnaires about the nutrients they consumed and to follow the recommended diet. Additionally, an examination of the questionnaires revealed that there were no changes in ω 3 PUFA intake from the diet throughout the intervention. Participants were advised not to use caffeine and alcohol 48 h prior to the experimental protocol.

All the tests were conducted at the Biochemistry Laboratory and the Laboratory of Human Functional Research. Echocardiographic examinations were performed at the Department of Cardiology, Clinical Hospital. The study protocol was approved by the Local Bioethics Commission (decision no. KBN 9.2016).

### Measures

#### Echocardiography and Blood Flow Measurements

All participants underwent one-dimensional M-mode, two-dimensional (2-D), and Doppler echocardiography at baseline (GE Vivid E9 with a 2.5-MHz transducer, General Electric, Horten, Norway) and after supplementation to determine their left ventricular mass and the left ventricular mass index (Devereux and Reichek, 1977). Left ventricular systolic function was assessed based on the measurements of the ventricular rejection fraction. To assess the vascular system function, the brachial artery diameter and dilation (7–12 MHz Logic 7, GE), arterial blood pressure (HEM– 907, Omron Corporation, Japan), and pulse wave velocity (AtCor Medcal, Australia) were measured.

#### Biochemical Tests

Blood samples were collected in the morning hours after an 8-h overnight fast, immediately after exercise, as well as 1 h and 24 h post-exercise from the cubital fossa vein. They were then left to clot at room temperature and centrifuged. The received serum was aliquoted, frozen at −80°C, and stored for further analysis.

To quantify the erythrocyte membrane content of fatty acids, the whole blood spot sample was discarded and then a drop was placed onto a spot card pre-treated with an antioxidant blend to prevent oxidative loss of PUFA. Blood spot cards were dried at room temperature in the dark for two weeks and subsequently stored in a −80°C freezer until shipment. Samples were sent in batches to a clinical laboratory (the Centro Diagnostico Delta, Italy) for analysis. The ω 3 PUFA index and erythrocyte membrane content of fatty acids were calculated using a dry blood drop method, a normalized method HS-Omega-3 Index®, gas chromatography GC-FID with a flame-ionisation detector (Centro Diagnostico Delta, Italy; certificate no. 8449/2010).

Cardiac damage markers, i.e., high-sensitivity cardiac troponin (hsTnT) and cardiac fatty acid binding protein (H-FABP) were assayed using immunoenzymatic methods (SED22HU 96 Tests, Cloud-Clone Corporation, Houston, TX, USA and Human H-FABP HK401 Elisa Kit, Hycult Biotech, Uden, the Netherlands, respectively), and creatine kinase MB (CKMB) was assayed by an immunoinhibition method (BioMaxima, Lublin, Poland).

In order to assay inflammatory cytokines, immunoenzymatic methods were applied; Human IL-6 High Sensitive ELISA kit, Diacone, France, for interleukin 6 (IL-6), and TNF-α-EASIA KAP1751, DIAsource, Belgium, for tumour necrosis factor alpha (TNF-α).

The lipid profile components (the serum concentration of total cholesterol, triglycerides (TG), low density lipoprotein cholesterol (LDL-C) and high density lipoprotein cholesterol (HDL-C) were assayed using enzymatic methods and a clinical chemical analyser RA-XT (Technicon Instruments Corporation, USA). The index of hemoconcentration (%) was obtained by calculating the difference between the highest and the lowest level of hematocrit and multiplying it by 100; the values of all biochemical variables were adjusted for changes in plasma volume.

All biochemical assays were carried out in a certified laboratory of the PN EN-ISO 9001: 2009 standard (certificate no. 129/2015), and per the instructions of assay manufacturers.

### Design and Procedures

Participants were tested on three separate sessions at the same time of the day (08.00–11.00 am). The first session consisted of a graded-exercise test to volitional exhaustion for determination of participants’ aerobic capacity (VO_2max_), heart rate (HR) and peak power output (W). All variables were determined to calculate the individual workloads for the eccentric exercise tests. VO_2max_ was measured with a cardiopulmonary exercise test system (CareFusion Germany 234 GMBH, Hoechberg Jaeger, Germany) on a mechanical treadmill (LE 200, Jaeger, Frankfurt, Germany). A summary of participants’ oxygen capacity and training status characteristics is provided in Table 1.

In the subsequent two sessions conducted immediately before supplementation or the placebo (pre-suppl vs. pre-placebo) and at the end of supplementation or the placebo phase (post-suppl vs. post-placebo), participants completed an eccentric exercise protocol (downhill running). All participants performed 30-min downhill running on a treadmill modified to run in reverse at a −16% grade. The test started with a velocity of 6 km/h and after 3 min, the velocity was increased until the HR corresponding to 70% VO_2max_ was reached. This protocol has been previously suggested to elicit a significant degree of muscle damage following a downhill run (Eston et al., 1995; Sorichter et al., 1997).

### Supplementation

For three weeks, participants consumed daily either six capsules (three in the morning and three in the evening) of concentrated fish oil containing 3,000 mg of ω 3 PUFA (BergenOmega-3; Natural Pharmaceuticals, Warsaw) or six gelatine capsules (three in the morning and three in the evening) prepared by the same producer. The dose in the supplGr was determined based on the results of a study by Jouris et al. (2011). One fish oil capsule contained 450 mg of ω 3 PUFA (142 mg of EPA and 267 mg of DHA). The extract was prepared specifically for this study. The energy content of 100 g of BergenOmega-3 was about 736 kcal. One gelatin capsule contained 1.3 g of lactose monohydrate instead of fish oil extract.

#### Statistical Analysis

After completing descriptive statistics of the collected variables, the Shapiro-Wilk test was performed for verification of data normality. The calculated descriptive statistics are presented as means and standard deviations (a mean ± SD). Analysis of variance (ANOVA) with repeated measures was used to compare the variables related to exercise (pre- and post-exercise) and supplementation (pre-suppl vs. post-suppl and pre-placebo vs. post-placebo). The significance of the between-group differences was evaluated by the Student-Newman-Keuls test. The data were analysed using the Statistical Package version 12 (StatSoft, Tulsa, OK, USA) and significance was set at *p* < 0.05.

## Results

### Basic Anthropometric, Echocardiography and Blood Flow Measurements

At baseline, there were no significant differences in all measured somatic variables (e.g., age, BMI, and body composition) and aerobic performance (VO_2max_, peak power, HR_max_) between athletes in the GrP and the GrSuppl (Table 1).

Echocardiographic heart variables were within the normal range in the GrSuppl and the control group (GrP). No significant differences were found in the LVM, LVMI and IVSDd between the groups, neither at baseline nor post supplementation (Table 2). A tendency for higher LVM, LVEF and pulse wave velocity in the post-suppl vs. pre-suppl was reported in the GrSuppl. No significant differences were found in the HR, BAD and FMD between the groups or in response to the supplementation procedure (Table 2).

**Table 2:** *E*chocardiography variables and blood flow measurements in the supplemented (GrSuppl) and the placebo group (GrP) before (pre-suppl vs. pre-placebo) and after (post-suppl vs. post-placebo) dietary intervention (mean ± SD).

Variables	GrSuppl (n = 12)		GrP (n = 12)		*p*
	Pre-suppl	Post-suppl	Pre-placebo	Post-placebo	Post-suppl vs. post-placebo
LVM (g)	219.0 ± 24.5	226.0 ± 25.5	*231.8 ± 38.2*	*228.0 ± 34.5*	ns
LVMI (g/m^2^)	116.0 ± 8.6	116.0 ± 10.8	*122.0 ± 11.2*	*120.0 ± 18.0*	ns
IVSDd (mm)	10.0 ± 1.3	10.6 ± 1.4	*11.3 ± 0.6*	*11.4 ± 1.4*	ns
LVPWTd (mm)	9.4 ± 1.1	9.9 ± 1.2	*10.6 ± 0.7*	*11.0 ± 1.2*	ns
LVEF %	62.6 ± 4.9	63.9 ± 4.8	*64.6 ± 8.5*	*64.4 ± 5.5*	ns
HR [b/min]	58.0 ± 8.0	56.0 ± 10.0	57.0 ± 8.0	58.0 ± 6.0	ns
PWV (m•s^−1^)	5.9 ± 1.1	6.3 ± 1.5	6.0 ± 1.3	5.8 ± 0.8	ns
BAD (mm)	4.1 ± 0.6	4.3 ± 0.6	4.3 ± 0.4	4.2 ± 0.7	ns
FMD (mm)	5.0 ± 0.5	5.0 ± 0.6	5.2 ± 0.3	5.0 ± 0.6	ns

LVM: left ventricular mass; LVMI: left ventricular mass index; IVSDd: interventricular septal thickness *(in diastole)*, LVPWTd: **left** ventricular posterior wall thickness in diastole; LVEF: left ventricular ejection fraction, HR: heart rate, PWV: pulse wave velocity; BAD: brachial artery diameter, FMD: flow-mediated vasodilation

### Erythrocyte Membrane Fatty Acids (Omega 3 Index) Profile

The values of the Omega 3 Index and erythrocyte membrane content of omega 3 fatty acids (ω 3 PUFA), EPA, DHA, omega 6 fatty acids (ω 6 FA), AA, and the AA/EPA ratio are presented in Table 3. The value of the Omega-3 Index in the GrSuppl increased significantly in response to supplementation (*p* < 0.001), and it was significantly greater in the GrSuppl compared to the GrP (*p* < 0.01). The erythrocyte membrane content of EPA and DHA in the GrSuppl was significantly higher at the completion of supplementation (*p* < 0.001 and *p* < 0.01, respectively). The post-suppl AA/EPA ratio was significantly lower than at baseline (*p* <0.001) in the GrSuppl and compared to the GrP (*p* < 0.05). The omega 6 fatty acid profile ω-6 FA was not significantly affected by supplementation (Table 3).

**Table 3:** The Omega-3 Index and erythrocyte membrane content of fatty acids in the supplemented group (GrSuppl) and the placebo group (GrP) before (pre-suppl vs. pre-placebo) and after (post-suppl vs. post-placebo) dietary intervention (mean ± SD).

Variables	GrSuppl (n = 12)		GrP (n = 12)		*p*
	Pre-suppl	Post-suppl	Pre-placebo	Post-placebo	Post-suppl vs. post-placebo
Omega-3 index [%]	3.9 ± 0.5	4.8 ± 0.8 ***	3.5 ± 0.3	3.9 ± 0.4	0.01
EPA [%]	0.4 ± 0.2	0.8 ± 0.3***	0.5 ± 0.1	0.7 ± 0.1	ns
DHA [%]	2.5 ± 0.6	2.9 ± 0.6**	2.0 ± 0.3	2.1 ± 0.4	0.05
AA/EPA	17.7 ± 6.5	8.1 ± 2.4***	14.2 ± 3.4	12.0 ± 3.7	0.05
ω 6 FA [%]	28.7 ± 2.8	27.4 ± 2.4	28.8 ± 3.1	28.9 ± 3.5	ns
AA [%]	6.6 ± 1.1	6.1 ± 1.2	7.3 ± 1.0	7.5 ± 1.0	0.05

Omega-3 index: the fatty acid index (ω3**)**, EPA: eicosapentaenoic acid; DHA: docosahexaenoic acid; AA/EPA: arachidonic acid to eicosapentaenoic acid ratio; ω-6 FA: ω-6 fatty acids; AA: arachidonic acid. Significant differences between measurements taken before and after supplementation: ** p < 0.01, *** p < 0.001

### Cardiac Markers and Cytokines

The statistical analysis revealed supplementation (suppl) and interaction (supplementation and exercise; suppl Ex) effects on the concentration of hsTnT (F = 6.9; *p* < 0.001 and F = 3.9; *p* < 0.015, respectively) (Table 4). In the GrSuppl, hsTnT levels were significantly lower immediately post-exercise and after 1 h of post-exercise recovery compared to the pre-supplementation levels (2.7 ± 0.9 vs. 5.1 ± 1.4 pg/ml, *p* < 0.05) and (2.9 ± 0.7 vs. 4.9 ± 0.8 pg/ml, *p* < 0.05), respectively (Table 5). Supplementation and exercise had an effect on the activity of CKMB (F = 6.7; *p* < 0.001) with only a tendency for lower CKMB activity after exercise in the GrSuppl compared to the GrP. The statistical analysis revealed a tendency for supplementation to influence the concentration of H-FABP (F = 3.9; *p* = 0.06). Its value in the GrSuppl measured after exercise (1 h and 24 h of recovery) was significantly lower than the pre-suppl (*p* < 0.05 and *p* < 0.05; respectively). The ω 3 PUFA supplement also had an effect on the concentration of VEGF (F = 129.0; *p* < 0.000) (Table 4). In the GrSuppl, the resting pre-suppl level of VEGF was significantly lower compared to the resting post-suppl levels (1.7 ± 0.6 vs. 2.7 ± 1.8 ng/ml; *p* < 0.05) and VEGF max (immediately after exercise) post-suppl was significantly lower compared to the VEGF max pre-suppl (1.7 ± 0.9 vs. 3.6 ± 1.5 ng/ml; *p* < 0.05). The effect of ω 3 PUFA supplementation on TNFα was also significant (F = 4.7; *p* < 0.05). The post-suppl TNFα levels at rest, in response to the exercise test and 1 h and 24 h post-exercise were significantly lower than before supplementation (*p* < 0.05, *p* < 0.01, *p* < 0.01, and *p* < 0.01, respectively). The post- suppl concentration of TNFα in the GrSuppl was also significantly lower than in the GrP (*p* < 0.05). There were no effects of supplementation or exercise on serum IL-6 levels (Table 5).

**Table 4:** The effect of supplementation with omega-3 fatty acids (suppl) and interaction supplementation with exercise (Suppl Ex) on cardiac damage markers *and cytokines* *(*F: Fisher’s F-ratio, *p: p*-value, η2: eta squared, α: effect size).

Variable	Effect	F	*p*	η^2^	α
hsTnT [pg/ml]	Suppl	6.9	*0.001*	*0.30*	*1.0*
	Suppl Ex	3.9	*0.015*	*0.14*	*0.8*
CKMB [U/l]	Suppl	0.2	*0.64*	*0.01*	*0.07*
	Suppl Ex	6.7	*0.001*	*0.24*	*1.0*
H-FABP [pg/ml]	Supl	3.9	*0.06*	*0.15*	*0.5*
	Suppl Ex	0.7	*0.58*	*0.03*	*0.2*
VEGF [ng/ml]	Supl	129.0	*0.000*	*0.85*	*1.0*
	Suppl Ex	0.21	*0.88*	*0.01*	*0.1*
TNF α [pg/ml]	Suppl	4.7	*0.05*	*1.18*	*0.6*
	Suppl Ex	0.6	*0.62*	*0.03*	*0.2*
IL-6 [pg/ml]	Suppl	0.4	*0.59*	*0.02*	*0.2*
	Suppl Ex	1.0	*0.32*	*0.02*	*0.2*

hsTN: high-sensitivity troponin, CKMB: creatine kinase isoenzyme MB, H-FABP: fatty acid binding protein, VEGF: vascular endothelial growth factor, TNFα: tumour necrosis factor alpha;

IL-6: Interleukin 6; Suppl: supplementation, SupplEx: supplementation and exercise

**Table 5:** Cardiac markers concentrations at rest, immediately after the cessation of graded eccentric exercise tests (max) and during the recovery period (1 h and 24 h post-exercise) in the supplemented group (GrSuppl) and the placebo group (GrP) before (pre-suppl vs. pre-placebo) and after (post-suppl vs. post-placebo) dietary intervention (mean ± SD).

Variables	GrSuppl (n = 12)		GrP (n = 12)		*p*
	Pre-suppl	Post-suppl	Pre-placebo	Post-placebo	Post suppl vs. post placebo
hsTnT [pg/ml]					
Rest	3.6 ± 1.2	2.0 ± 1.2	6.9 ± 1.9	5.6 ± 2.2	ns
Max	5.1 ± 1.4	2.7 ± 0.9	12.0 ± 4.4	5.3 ± 2.1	ns
1 h post-exercise	4.9 ± 0.8	2.9 ± 0.7	4.4 ± 0.8	4.7 ± 0.8	*p* < 0.05
24 h post-exercise	6.3 ± 1.2	3.7 ± 0.5	4.7 ± 1.2	3.1 ± 0.5	ns
CKMB [U/l]					
Rest	11.5 ± 2.8	11.7 ± 5.1	13.9 ± 5.9	10.5 ± 3.8	ns
Max	14.2 ± 7.6	19.6 ± 10.1	18.7 ± 4.6	21.7 ± 13.1	ns
1 h post-exercise	12.7 ± 5.1	16.8 ± 7.8	18.7 ± 4.4	22.8 ± 12.2	ns
24 h post-exercise	22.4 ± 8.2	21.1 ± 11.0	18.9 ± 4.4	15.8 ± 3.0	ns
H-FABP [ng/ml]					
Rest	4.3 ± 1.6	3.4 ± 1.6	3.7 ± 0.9	3.0 ± 1.4	ns
Max	6.9 ± 3.5	5.1 ± 3.4	6.5 ± 3.6	5.1 ± 2.2	ns
1 h post-exercise	5.0 ± 3.2	3.9 ± 2.0 *	3.9 ± 2.0	3.1 ± 0.9	ns
24 h post-exercise	5.3 ± 3.1	4.1 ± 2.1 *	3.6 ± 1.2	3.2 ± 1.4	ns
VEGF [ng/ml]					
Rest	2.7 ± 1.8	1.7 ± 0.6 *	4.0 ± 1.8	3.7 ± 2.2	ns
Max	3.6 ± 1.5	1.7 ± 0.9 *	5.1 ± 2.4	7.4 ± 4.4	ns
1 h post-exercise	3.3 ± 2.2	1.5 ± 1.0	4.0 ± 1.3	8.4 ± 5.0	ns
24 h post-exercise	4.0 ± 1.9	1.5 ± 0.8 **	3.9 ± 1.4	1.9 ± 0.7	ns

hsTNT: high-sensitivity troponin, CKMB: creatine kinase isoenzyme MB, H-FABP: fatty acid binding protein, VEGF: vascular endothelial growth factor. Significant differences between measurements taken before and after supplementation: * p < 0.05, ** p < 0.01

### The Effect of ω 3 PUFA on the Lipid Profile

The analysis of variance revealed a significant effect of supplementation on serum adiponectin levels (F = 22.7; *p* < 0.000) and adiponectin was significantly elevated after supplementation compared to the pre-suppl concentrations. Serum leptin decreased significantly in response to supplementation compared to the pre-suppl concentrations (Table 6). A significant effect of ω 3 PUFA supplementation on the serum levels of HDL-C and TG was observed (F = 11.1, *p* < 0.003 and F = 35.0, *p* < 0.001, respectively). The baseline concentration of HDL-C in the GrSuppl increased significantly in response to supplementation (68.9 ± 13.0 vs. 57.6 ± 12.3 mg/dl, *p* < 0.05). LDL-C did not change significantly in response to supplementation, but its basal concentration in the GrSuppl at week 3 tended to be lower than at pre-supplementation. The GrSuppl’ concentration of TG measured after supplementation was significantly lower when compared to the baseline (103.0 ± 25.4 vs. 118.0 ± 27.1, *p* < 0.05). The effects of ω 3 PUFA supplementation on the lipid profile components are presented in Tables 6 and 7.

**Table 6:** The effect of supplementation with omega-3 fatty acids (suppl) and interaction supplementation with exercise (Suppl Ex) on the lipid profile components (F: Fisher’s F-ratio, *p: p*-value, η2: eta squared, α: effect size).

Variables	Effect	F	*p*	η^2^	α
Cholesterol l [mg/dl]	Suppl	0.7	*0.420*	*0.03*	*0.1*
	Suppl Ex	2.1	*0.100*	*0.09*	*0.5*
HDL [mg/dl]	Suppl	11.1	*0.003*	*0.34*	*0.9*
	Suppl Ex	3.8	*0.010*	*0.15*	*0.8*
LDL [mg/dl]	Suppl	0.4	*0.530*	*0.02*	*0.1*
	Suppl Ex	1.2	*0.310*	*0.05*	*0.3*
TG [mg/dl]	Suppl	35.0	*0.000*	*0.61*	*1.0*
	Suppl Ex	0.4	*0.740*	*0.02*	*0.1*

HDL: high-density lipoprotein, LDL: low-density lipoprotein, TG: triglycerides, Suppl: supplementation, Suppl Ex: supplementation and exercise.

**Table 7:** Adiponectin, leptin, and cytokine concentrations at rest, immediately after the cessation of graded eccentric exercise tests (max) and during the recovery period (1 h and 24 h post-exercise) in the supplemented group (GrSuppl) and the placebo group (GrP) before (pre-suppl vs. pre-placebo) and after (post-suppl vs. post-placebo) dietary intervention (mean ± SD).

Variables	GrSuppl (n = 12)		GrP (n = 12)		*p*
	Pre-suppl	Post-suppl	Pre-placebo	Post-placebo	Post suppl vs. post placebo
Adiponectin [µg/ml]					
Rest	23.8 ± 8.5	31.4 ± 7.7**	28.8 ± 8.5	30.1 ± 13.5	ns
Max	25.0 ± 8.1	37.1 ± 12.0***	32.1 ± 9.9	33.1 ± 12.9	ns
1 h post-exercise	20.2 ± 8.8	33.2 ± 9.6***	37.8 ± 15.1	37.8 ± 13.4	ns
24 h post-exercise	22.9 ± 5.2	34.1 ± 8.9***	31.0 ± 11.1	38.0 ± 10.2	ns
Leptin [ng/ml]					
Rest	3.3 ± 1.9	2.7 ± 1.0 **	2.6 ± 0.4	2.7 ± 0.3	ns
Max	3.5 ± 2.0	2.9 ± 1.1**	2.8 ± 0.7	2.9 ± 0.8	ns
1 h post-exercise	3.1 ± 1.8	2.3 ± 0.7***	2.5 ± 0.5	2.5 ± 0.6	ns
24 post-exercise	3.3 ± 1.5	2.5 ± 0.8***	2.8 ± 0.8	2.9 ± 0.7	ns
TNFα [pg/ml]					
Rest	9.7 ± 2.7	5.6 ± 2.6*	13.7 ± 7.4	12.5 ± 2.4	0.05
Max	24.0 ± 15.2	10.5 ± 4.4 **	22.9 ± 13.8	22.7 ± 17.4	ns
1 h post-exercise	21.9 ± 16.8	8.4 ± 3.7 **	18.7 ± 11.4	21.3 ± 12.0	ns
24 h post-exercise	19.8 ± 14.2	11.6 ± 5.5	13.9 ± 6.5	13.7 ± 7.3	ns
IL-6 [pg/ml]					
Rest	1.4 ± 1.3	1.9 ± 1.8	1.5 ± 1.3	2.2 ± 2.0	ns
Max	2.0 ± 1.9	1.7 ± 1.0	2.7 ± 1.5	2.5 ± 2.3	ns
1 h post-exercise	2.7 ± 2.3	2.3 ± 1.3	3.1 ± 2.0	3.0 ± 1.9	ns
24 h post-exercise	1.8 ± 1.2	1.0 ± 0.9	2.0 ± 1.2	2.4 ± 1.6	ns

TNFα: tumour necrosis factor alpha; IL-6: Interleukin 6. Significant differences between measurements taken before and after supplementation at * p < 0.05, ** p < 0.01, *** p < 0.001

## Discussion

In the present study, the effects of three-week dietary supplementation with ω 3 PUFA on the content of ω 3 PUFA in the erythrocyte cell membrane, cardiovascular function, serum concentration of cardiac markers, the lipid profile, and pro-inflammatory cytokines before and after downhill running in long distance runners were evaluated.

The Omega-3 Index determines the percentage of EPA and DHA in relation to all fatty acids of the red blood cells membrane (Harris et al., 2017; Schacky, 2020). All examined athletes had a low Omega-3 Index (below 4%) indicating a deficiency of ω 3 PUFA in the diet. A significant increase in the erythrocytes ω 3 PUFA (EPA and DHA) content and a significant decrease in AA content with a lower AA/EPA ratio in response to supplementation are important findings of the present study. Despite the observed benefits, the expected effect of achieving the recommended Omega-3 Index value (> 8 %) has not been accomplished (Bilinski et al., 2020). The ω 3 PUFAs are an essential part of the cell membrane structure and support important physiological functions, therefore, it is crucial to ensure that the diet of endurance athletes contains sufficient amounts of EPA and DHA.

Low levels of Omega-3 Index (4.97 ±1.19 %) have been demonstrated in a group of 106 elite endurance-trained athletes (Schacky et al., 2014). It can be assumed that in physically active and healthy individuals, 6–8-week (Thielecke et al., 2020) or even 12-week (Biliński et al., 2020) supplementation with ω 3 PUFA would increase their Omega-3 index to the desired levels (> 8%). This seems to be especially important for long distance runners who are at a higher risk of SCD and excessive inflammatory reaction associated with an increased AA erythrocytes content and a higher AA/EPA ratio in the erythrocytes after marathon running (Davinelli et al., 2019). In the present study, the AA/EPA ratio decreased significantly in response to ω 3 PUFA supplementation. The lower AA/EPA ratio may indicate a lower risk of coronary events and the anti-inflammatory effect of the ω 3 PUFA protocol used in this study (Nelson and Raskin, 2019).

## ω 3 PUFA and Cardiac Biomarkers

Another significant result of the study is the reduction of serum concentration of certain myocardial damage markers (i.e., hsTN and CKMB) after the downhill running exercise protocol in the ω 3 PUFA supplemented group. Elevated (> 14 ng/l) concentrations of cardiac troponin after completion of a marathon race have been previously documented (Mielgo-Ayuso et al., 2020; Rubio-Arias et al., 2020). Also, an increase of high sensitivity TN (hsTnT) range from 69.8% to 100% has been demonstrated in marathon runners (Leckie et al., 2019; Richardson et al., 2018).

The increased levels of cardiac-specific markers, such as hsTnT, CKMB, and H-FABP have been commonly observed in athletes immediately after prolonged running and have been shown to be a result of excessive hemodynamic stress on the heart and/or hypoxia (Stavroulakis and George, 2020; Żebrowska et al., 2019). The markers’ presence in the blood indicate myocardial cell damage and H-FABP is considered to be the most sensitive cardiac biomarker, that enables detection of an early stage myocardial damage and/or ischemia (Raj Kulshrestha et al., 2022). The mechanisms responsible for the release of cardiac biomarkers into the blood during exercise are not fully understood (Mahanty and Xi, 2020). It is hypothesized that shear stress associated with exercise makes the cardiomyocyte cell membrane more permeable, allowing the release of cardiac markers into the blood (Kaleta Duss et al., 2020; Knebel et al., 2009), but, cardiomyocyte damage due to hypoxia and ischemia cannot be ruled out. A prolonged elevation of the cardiac markers after strenuous exercise may suggest coronary atherosclerosis and increased cardiac risk (Cunningham et al., 2017; Predel, 2014). On the other hand, lower concentration of the specific cardiomyocyte damage markers after a marathon run or long-term endurance exercise may confirm the increased resistance to ischemic myocardial damage. In the present study, we demonstrated that ω 3 PUFA supplementation increased the ω 3 content in the erythrocyte cell membrane and lowered the AA/EPA ratio and hsTnT release during eccentric exercise. These findings are consistent with previous reports on the negative correlation between the supply of ω 3 PUFA in the diet and the hsTnT concentration in recreational marathon runners (Mielgo-Ayusoi et al., 2020).

Supplementation also had an effect on the baseline VEGF levels. VEGF belongs to a group of signalling proteins regulating the permeability of blood vessel walls and it is a crucial factor initiating the process of angiogenesis (Prior et al., 2004). Increased binding of VEGF to VEGF receptors of the endothelium may affect the activation of angiogenesis in skeletal muscles, which could improve the process of adaptation to training. However, the mechanisms of cardiovascular protection and benefits of ω 3 PUFA for athletes are still under investigation (Kolar et al., 2021). A study by Zhuang et al. (2013) on a model of human endothelial cells demonstrated that VEGF production was lower following ω 3 PUFA supplementation. The ω 3 PUFAs have been shown to decrease sprouting angiogenesis by suppressing VEGF-stimulated endothelial cell proliferation. Determining the effects of EPA and DHA on VEGF serum concentration changes and related effects on the vascular system in healthy individuals subjected to exercise training requires further investigation.

In our study, ω 3 PUFA supplementation had a beneficial effect on the myocardium biomarker levels and a potential protective effect on the cardiovascular system due to the reduction of the exercise-induced overload of the cardiovascular system. Moreover, ω 3 PUFA could beneficially modulate the lipid profile via enzymatic reactions, and have an impact on the key endothelial mediators (nitric oxide, cyclooxygenase and cytochrome P450) (Burillo et al., 2012; Oppedisano et al., 2020; Żebrowska et al., 2014), thus improving the endothelial function. The resting left ventricular systolic function and the function of the vascular system based on the brachial artery diameter, arterial blood pressure, and pulse wave velocity did not change significantly following supplementation. Therefore, future research should establish the potential mechanisms mediating interactions between ω 3 PUFA, exercise and cardiovascular adaptation in athletes.

### Omega 3 PUFA and Cytokines and the Lipid Profile

Our findings indicate that three-week supplementation with ω 3 PUFA had a positive effect on TNFα, of which serum levels at rest and after eccentric exercise were significantly reduced when compared to the pre-suppl levels. Moreover, the increased adipocytokine, i.e., adiponectin production, seemed to have a positive effect on the blood lipid profile and inflammation (Yanai et al., 2019). Similarly, recent human studies revealed that ω 3 PUFA supplementation for a period of 12–24 weeks increased levels of adiponectin and reduced TNFα concentrations (Becic et al., 2018) in subjects with prediabetes. The results of our study are also consistent with the observations of Tartibian et al. (2011) who reported lower TNFα concentrations and CK activity 24 h and 48 h after eccentric exercise in response to short-term ω 3 PUFA supplementation. In another study, a significant reduction in the concentration of TNFα after eccentric exercise and decreased CK activity were observed in response to a diet rich in ω 3 PUFA lipid extract at a dose of 1200 mg daily for 26 days (Mickleborough et al., 2015). The positive effects of ω 3 PUFA supplementation on TNFα were also observed in sedentary individuals, however, the decrease in the TNFα level was smaller than in the group of athletes (Buonocore et al., 2020).

In our study, a beneficial effect of ω 3 PUFA supplementation was revealed when higher adiponectin and HDL cholesterol concentrations were recorded. It has been previously presented that adiponectin increases fatty acid oxidation in skeletal muscle cells through the activation of AMP-activated protein kinase (AMPK) and the mitogen-activated protein kinase (MAPK) signalling pathways. Additionally, the concentration of HDL cholesterol was significantly higher in the supplemented group compared to the pre-supplementation levels. Moreover, significantly lower values of TG and a trend for lower LDL cholesterol levels were also observed after ω-3 PUFA diet. It has been shown that ω 3 PUFAs improve the anti-atherogenic profile of the HDL cholesterol fraction, and this is associated with an increase in the levels of larger particles of the HDL2 subfraction and a decrease in the concentration of smaller particles of the HDL3 subfraction (Burillo et al., 2012; Ines and Cadler, 2018). The concentration of the HDL2 has an inverse relationship with the cardiovascular risk, while the concentration of HDL3 shows a direct relationship (Camont et al., 2011). It has been reported that ω 3 PUFA supplementation can reduce TG concentrations by approximately 15% (Abdelhamid et al., 2020) and with a parallel increase in HDL levels, it can decrease the risk of cardiovascular disease.

The results of our study confirm previous observations of the inhibitory effect of adiponectin on inflammation via reducing the expression of pro-inflammatory cytokines (Choi et al., 2020), improvement in the lipid profile and protective effect on the cardiovascular system in distance runners (Innes and Calder, 2020; Tartibian et al., 2011). Nevertheless, there are some limitations of the study that should be addressed. The study included distance runners who consumed either the placebo or the ω-3 PUFA diet for three weeks. Further investigations with a longer supplementation period could provide more specific results and enable generalization of the study findings.

## Conclusions

To summarise, the increased erythrocyte membrane EPA and DHA content and lower blood concentrations of the cardiac damage markers and inflammation mediators measured after downhill running in distance runners supplemented for three weeks with ω 3 PUFA suggest that their cardiovascular function and regeneration capacity after the eccentric muscle damaging exercise protocols were improved.
